# Changes in plasma fatty acid composition in females with lipedema following low-carbohydrate vs low-fat diets and associations with pain reduction

**DOI:** 10.1186/s12937-026-01304-y

**Published:** 2026-03-11

**Authors:** Julianne Lundanes, Vilde Fiske Nes, Patrik Hansson, Rikard Fristedt, Rikard Landberg, Catia Martins, Siren Nymo

**Affiliations:** 1 Department of Clinical and Molecular Medicine, Faculty of Medicine and Health Sciences, Norwegian University of Science and Technology (NTNU), Forsyningssenteret, Prinsesse Kristinas gate 5, Trondheim, 7030 Norway; 2 Clinic of Surgery, Nord-Trøndelag Hospital Trust, Namsos Hospital, Namsos, Norway; 3 Clinical Nutrition Research Group, Department of Clinical Medicine, UiT The Arctic University of Norway, Tromsø, Norway; 4 Department of Food and Nutrition and Sport Science, University of Gothenburg, Gothenburg, Sweden; 5 Division of Food and Nutrition Science, Department of Life Sciences, Chalmers University of Technology, Gothenburg, Sweden; 6 Department of Nutrition Sciences, University of Alabama at Birmingham (UAB), Birmingham, AL USA; 7 Department of Public Health and Nursing, Faculty of Medicine and Health Sciences, Norwegian University of Science and Technology (NTNU), Trondheim, Norway

**Keywords:** Omega-3, Omega-6, Inflammation, Obesity

## Abstract

**Background:**

Lipedema is a chronic female disease characterized by a painful accumulation of adipose tissue in the limbs. Plasma fatty acid (FA) composition has been proposed as a potential modulator of pain. However, the pathophysiology behind lipedema pain remains uncertain. The primary objective of this secondary analysis was to compare changes in plasma concentrations of FAs between low-energy diets either low in carbohydrates or low in fat, in females with lipedema and obesity. A secondary objective was to investigate potential associations between changes in pain and changes in the concentration of several FAs.

**Methods:**

Females with lipedema and obesity (BMI 30–45 kg/m^2^) were randomized to isocaloric low-energy diets, either low-carbohydrate diet (LCD) or low-fat diet for 8 weeks. Plasma concentrations of FAs were quantified using gas chromatography and subjective pain using the Brief Pain Inventory, before and after the intervention.

**Results:**

70 females were included in the analyses, with a mean BMI of 37 ± 5 kg/m^2^ and mean age of 47 ± 11 years. Significant decreases in the concentration of the saturated FAs (SFAs) myristic, stearic, and behenic acids, and the polyunsaturated FAs (PUFA) gamma-linolenic (GLA), dihomo-gamma-linolenic (DGLA), alpha-linolenic (ALA), eicosapentaenoic (EPA), and docosapentaenoic acids (DPA) were seen in both groups. A reduction in the SFA arachidic acid, and the monounsaturated FAs (MUFA) palmitoleic and oleic acids was seen in the LCD group only, while an increase in the SFA lignoceric acid and a decrease in the PUFA linoleic acid was seen only the low-fat diet group. Changes in myristic and palmitic acids (SFAs) were positively associated with changes in pain.

**Conclusion:**

A reduction in most FAs was found after energy restricted LCD and low-fat diets in females with lipedema and obesity. Notably, reductions in SFAs seem to be associated with the reduction in pain seen in the LCD group, especially myristic acid. These findings suggest that FA composition may play a role in pain reduction in females with lipedema.

**Trial registration:**

NCT04632810, Effect of Ketosis on Pain and Quality of Life in Patients With Lipedema (Lipodiet).

**Supplementary Information:**

The online version contains supplementary material available at 10.1186/s12937-026-01304-y.

## Introduction

Lipedema is a chronic female disease characterized by a painful accumulation of adipose tissue in the limbs [1]. Females with lipedema report the lipedema associated pain as dull, oppressive, stabbing, pulling and burning [2]. The pathophysiology behind lipedema pain remains uncertain, however, it has been suggested to be due to inflammation and hypoxia [3], nociceptive pain due to tissue damage and allodynia [4, 5], or a mechanism for pressure transmission caused by the fibrosis and increase in extracellular matrix [6].

Dietary interventions using low-carbohydrate diets (LCD) or ketogenic diets have shown potential to reduce pain in females with lipedema [7–10], although the underlying mechanisms remains unclear. Several hypotheses have been suggested [11], and one plausible mechanism is reduction in inflammation. However, no direct association between inflammatory markers and pain reduction were found in a population with lipedema and obesity following a low-carbohydrate diet [12], hence other mechanisms may play a role.

Fatty acid (FA) composition has been proposed as a potential modulator of pain [13, 14]. FA composition may modulate pain indirectly through reduction in inflammation. Omega-3 FAs have the potential to reduce inflammation [15], whereas omega-6 FAs may increase inflammation, although the existing evidence is inconsistent [16]. Omega-3 FAs have been shown to alleviate pain sensations associated with several chronic pain conditions, such as rheumatoid arthritis, dysmenorrhea, and inflammatory bowel disease [17]. Saturated FAs (SFAs) have been positively associated with low-grade inflammation [18], and pain in animal studies [19]. Even though plasma FA composition will likely change with alterations in the macronutrient composition of the diet, in particular its fat content, intake of FAs is not always correlated with its plasma concentrations [20].

Therefore, the primary objective of this secondary analysis was to compare changes in plasma concentrations of FAs between two low-energy diets with different macronutrient composition – either a low-carbohydrate or a low-fat diet, in females with lipedema and obesity. A secondary objective was to investigate potential associations between changes in pain and changes in the plasma concentration of several FAs.

## Methods

### Study design

This paper consists of an explorative analysis of a randomized controlled trial (RCT) comparing a low-energy LCD to an isocaloric low-energy diet for 8 weeks in females with lipedema [7]. Approval was granted by the Regional Ethical Committee (REK 93888), and the study is registered in Clinicaltrials.gov (NCT04632810). All participants provided written, informed consent in accordance with the Helsinki Declaration prior to enrollment. Participants were randomized in a 1:1 ratio using block randomization with stratification based on BMI categories (30.0-34.9, 35.0-39.9, 40.0–44.9 kg/m^2^). Randomization was performed by a web-based randomization system developed and administered by the Faculty of Medicine and Health Sciences, Norwegian University of Science and Technology (NTNU), Trondheim, Norway. The data collection was performed using eFORSK, a web-based system developed and administered by Helse Midt-Norge IT (Central Norway Regional Health Authority’s IT department).

### Study population

This study included females aged 18 to 75 years, with a BMI of 30–45 kg/m^2^ [21, 22], with a diagnosis of lipedema and weight stable for the past three months (± 3 kg). The exclusion criteria included both acute and chronic kidney disease or failure, malignant or infectious disease, previous bariatric surgery, diabetes, psychiatric diseases, pregnancy, breastfeeding, use of medications known to affect body weight, insufficient proficiency in a Scandinavian language, and participation in another obesity or lipedema treatment program (except regular physiotherapy). No restrictions were placed on the use of compression garments/pulsators; thus, the participants who used these prior to the study were allowed to continue their use throughout the study period.

### Dietary intervention

The diets were matched for energy and protein, but differed in carbohydrates (CHO) and fat composition. Both diets consisted of 1200 kcal/day and 60 g of protein per day. The LCD consisted of 75 g (25 energy percentage (E%)) CHO and 73 g (55 E%) fat per day. The low-fat diet consisted of 180 g (60 E%) CHO and 27 g (20 E%) of fat per day.

### Compliance

The participants were followed weekly by a dietitian, either by phone or face-to-face, based on convenience. During these sessions, body weight was measured, ketosis was monitored (see ketosis section for details), potential side effects discussed, and dietary records reviewed, all with the aim of supporting adherence and minimizing dropout. Adjustments to the diets were made by the dietitian if needed, within the set parameters for energy intake and macronutrient distribution. Participants were instructed to complete daily food records throughout the diet period. These records were subsequently analyzed for intake of energy (kcal/day) and macronutrients (g/day, E%) using a web-based analysis tool [21] based on the Norwegian Food Composition Table (22). The fatty acid intake was collected from pre-coded food diaries. The pre-coded food diaries assess daily food intake with an additional photographic booklet that contains pictures of different portion sizes ranging from small to large. Scanning and calculation were performed by the University of Oslo (Department of Nutrition, Institute of Basal Medical Science). The pre-coded food diaries were scanned using the Cardiff TeleForm program version 10.5.1 (Datascan Oslo, Norway), and assessed with the food databases AE-18 and the Kostberegningsystem (KBS) calculation software system (version 7.4 at the Department of Nutrition, Institute of Basal Medical Sciences, University of Oslo). Absolute amounts in grams per day of total fats, SFAs, MUFAs, PUFAs, Omega-3 FAs, Omega-6 FAs, and Omega-6/Omega-3 ratio were collected.

### Outcome variables

The following variables were assessed at BL and after the diets (week 9) in the obesity outpatient clinic at St. Olavs University Hospital.

### Body weight

Weight in kilograms was measured with Seca 876 (SECA) to the nearest 0.1 kg.

### Pain

Pain was assessed using brief pain inventory. Only the question assessing pain now (How much pain are you in right now? ) was used in the present analysis. The brief pain inventory measures pain on a numeric rating scale from 0 to 10. All pain variables from this sample have been previously published [7].

### Fatty acid composition

Blood samples were taken in a fasting state, and 500 KIU of aprotinin (DSM, Coatech AB) per milliliter of whole blood was added to the EDTA tubes. The tubes were centrifuged at 2106 G at 18 °C for 10 min. The plasma was retrieved, and frozen at -80 °C until analysis. The samples were analyzed at the Department of Life Sciences, Division of Food and Nutrition Science at Chalmers University of Technology, in Gothenburg, Sweden. Total fatty acid analysis was done according to the method described in Stråvik et al. [23]. In brief, preparation of plasma samples included methylation/transesterification of FA and extraction of methyl ester FA (FAME) with n-hexane. The samples were analyzed using gas chromatography with flame ionization detector (GC-FID) that utilized a fast gas chromatography (GC) principle with He as the carrier gas and narrow bore capillary column.

Each sample batch was encompassed by study QC (quality control) samples pooled from the study samples and long-term in-house QC samples. Each batch was started by system suitability injections (SST), which was used to monitor the status of the instruments prior and post sample analysis. The NuCheck GLC463 standard was used and diluted to a 6-point external calibration curve that was used for quantitation. Methyl tricosanoate (23:0) was used as internal standard to account for sample preparation losses and matrix effects. The following FAs were quantified: Lauric acid (C12:0), myristic acid (C14:0), palmitic acid (C16:0), stearic acid (C18:0), arachidic acid (C20:0), behenic acid (C22:0), lignoceric acid (C24:0), myristoleic acid (C14:1), palmitoleic acid (PA; C16:1), oleic acid (OA; C18:1), eicosenoic acid (C20:1), nervonic acid (C24:1), linoleic acid (LA; C18:2), gamma-linolenic acid (GLA; C18:3n6), eicosadienoic acid (C20:2), dihomo-gamma-linolenic acid (DGLA; C20:3n6), arachidonic acid (AA; C20:4), adrenic acid (C22:4), alpha-linolenic acid (ALA; C18:3n3), eicosapentaenoic acid (EPA; C20:5n3), docosapentaenoic acid (DPA; C22:5n3), and docosahexaenoic acid (DHA; C22:6n3).

### Statistical analysis

Statistical analysis was performed using Stata (StataCorp. Release 19. College Station, TX, USA), and data presented as mean and standard deviation (SD) or estimated marginal means with a corresponding 95% confidence interval (CI). Residuals were checked for normality with Shapiro Wilk test and visual inspection of histograms. Statistical significance was assumed at *P* < 0.01. Group differences in the changes from BL were estimated by linear mixed-effect models (LMM). The fixed part was specified in terms of two dummy variables: one for time and one for group differences (LCD versus low-fat diet) post intervention (W9), as the BL means can be assumed to be the same given the randomized design [24, 25]. The mean difference in changes from BL is equivalent to the estimated mean group difference post intervention. A random intercept for subject was included to account for within-subject variation. Multiple linear regression was performed to investigate associations between pain and FAs. The outcome is the individual-level change in pain (week 9 minus baseline), and the predictors include changes in each FA (week 9 minus baseline). We additionally adjusted for group and weight loss. Change variables, as well as the regression model residuals, were checked for normality using Shapiro Wilk test and visual inspection of histograms. Statistical significance was assumed at *P* < 0.05 after adjusting for False Discovery Rate for the regression models. Figures were generated using GraphPad Prism (Version 10.0.2 for Windows, GraphPad Software, Boston, Massachusetts, USA).

## Results

### Study participants

A total of 70 females were included in this analysis, with a mean age of 47.3 ± 10.9 years and a mean BMI of 36.9 ± 4.9 kg/m^2^, see Table 1. Changes in body weight and composition, as well as pain sensation, over time following the two dietary interventions have been previously published [7]. Briefly, the LCD group lost significantly more weight (-10.2 kg versus − 7.4 kg) and experienced a significantly greater reduction in pain (-1.3 versus − 0.2 on a numeric rating scale) compared with the low-fat diet group.

**Table 1:** Baseline characteristics of the participants

	All participants (*n* = 70)
Age, years	47.3 ± 10.9
Height, cm	167.2 ± 6.1
BMI, kg/m^2^	36.9 ± 4.9
Weight, kg	103.2 ± 14.6

Data presented as mean ± standard deviation (SD). *LCD* Low carbohydrate diet, *BMI* Body mass index

### Fatty acid composition

Dietary intake of FAs is presented in Supplementary Table 1. Both diet groups reported a decrease in total fat, SFAs and MUFAs, while only the low-fat diet group had a decrease in PUFAs, Omega-3 FAs and Omega-6 FAs. There was a significant larger reduction in the low-fat diet group for total fats, MUFAs, PUFAs, Omega-3, and Omega-6 FAs. No change or difference between groups was seen for the omega-6 to omega-3 ratio.

Plasma concentrations of the SFAs before and after the two diets, as well as changes within groups and differences between groups are presented in Table 2. A significant decrease was seen in both the LCD and low-fat diet groups for myristic acid (-11.24 µg/mL and − 5.24 µg/mL, respectively), stearic acid (-52.24 µg/mL and − 36.23 µg/mL, respectively), and behenic acid (-9.76 µg/mL and 8.80 µg/mL, respectively). Only the LCD group had a reduction in arachidic acid (-1.54 µg/mL), while only in the low-fat diet group had an increase in lignoceric acid (2.9 µg/mL). The LCD group had a significantly larger reduction in myristic acid (-6.00 µg/mL) and arachidic acid (-1.35 µg/mL) compared to the low-fat diet group, and the low-fat diet had a larger increase in lignoceric acid (3.01 µg/mL) compared to the LCD group.

**Table 2:** Plasma concentrations of fatty acids before and after low-carbohydrate or low-fat diets and changes within and between groups

	Baseline	Week 9	Change from baseline to week 9			Difference in change between groups		
	Mean(SD)	Mean (SD)	EMM	95% CI	*P* value	EMM	95% CI	*P value*
Saturated fatty acids (SFA)								
Lauric acid, C12:0, µg/ml								
LCD	3.41 (1.80)	2.97 (1.45)	-0.63	-1.26 to -0.00	0.048	0.04	-0.78 to 0.86	0.925
Low-fat diet	3.33 (2.04)	2.80 (1.60)	-0.67	-1.28 to -0.06	0.032			
Myristic acid, C14:0, µg/ml								
LCD	23.93 (6.90)	12.93 (4.61)	-11.24	-14.22 to -8.25	**< 0.001**	-6.00	-9.90 to -2.09	**0.003**
Low-fat diet	24.15 (10.72)	18.68 (8.72)	-5.24	-8.37 to -2.11	**0.001**			
Palmitic acid, C16:0, µg/ml								
LCD	539.00 (97.02)	495.12 (75.55)	-44.35	-80.57 to -8.12	0.016	-27.98	-76.17 to 20.22	0.255
Low-fat diet	533.66 (132.66)	514.24 (109.56)	-16.37	-54.06 to 21.32	0.395			
Stearic acid, C18:0, µg/ml								
LCD	195.12 (40.0)	143.38 (30.34)	-52.24	-63.82 to -40.66	**< 0.001**	-16.00	-31.74 to -0.27	0.046
Low-fat diet	189.80 (38.18)	153.53 (39.98)	-36.23	-48.34 to -24.13	**< 0.001**			
Arachidic acid, C20:0, µg/ml								
LCD	6.21 (1.53)	4.41 (1.45)	-1.54	-2.28 to -0.80	**< 0.001**	-1.35	-2.34 to -0.35	**0.008**
Low-fat diet	5.27 (1.55)	5.32 (3.71)	-0.19	-0.97 to 0.59	0.630			
Behenic acid, C22:0, µg/ml								
LCD	26.15 (15.85)	16.21 (13.28)	-9.76	-14.15 to -5.35	**< 0.001**	-0.96	-6.88 to 4.59	0.751
Low-fat diet	23.9 (15.0)	16.0 (10.3)	-8.80	-13.33 to -4.27	**< 0.001**			
Lignoceric acid, C24:0, µg/ml								
LCD	16.81 (4.35)	16.82 (6.66)	-0.08	-1.66 to 1.51	0.926	-3.01	-5.21 to -0.81	**0.007**
Low-fat diet	16.26 (5.23)	19.15 (7.48)	2.93	1.28 to 4.59	**0.001**			
Monounsaturated fatty acids (MUFA)								
Myristoleic acid, C14:1, µg/ml								
LCD	1.92 (2.20)	2.21 (2.59)	-0.61	-1.12 to -0.86	0.019	-0.57	-1.23 to 0.10	0.094
Low-fat diet	2.59 (1.87)	2.45 (1.88)	-0.04	-0.48 to 0.40	0.859			
Palmitoleic acid (PA), C16:1, µg/ml								
LCD	46.53 (16.73)	32.05 (12.83)	-15.90	-21.99 to -9.81	**< 0.001**	-19.24	-27.49 to -10.99	**< 0.001**
Low-fat diet	50.46 (20.30)	52.09 (25.55)	3.34	-3.03 to 9.71	0.305			
Oleic acid (OA), C18:1, µg/ml								
LCD	587.84 (136.80)	508.51 (93.82)	-77.85	-122.39 to -33.30	**0.001**	-61.22	-121.24 to -1.20	0.046
Low-fat diet	573.04 (171.45)	552.55 (128.02)	-16.22	-63.28 to 30.02	0.485			
Eicosenoic acid, C20:1, µg/ml								
LCD	9.01 (20.42)	4.19 (1.73)	-2.79	-7.37 to 1.76	0.230	-0.14	-5.68 to 5.39	0.959
Low-fat diet	5.09 (4.32)	4.30 (2.10)	-2.64	-7.42 to 2.14	0.278			
Nervonic acid, C24:1, µg/ml								
LCD	19.75 (20.17)	18.43 (22.59)	-0.80	-5.23 to 3.63	0.723	-2.62	-8.80 to 3.63	0.407
Low-fat diet	16.30 (15.51)	17.92 (21.72)	1.82	-2.74 to 6.37	0.434			
Polyunsaturated fatty acids (PUFA)								
Omega-6								
Linoleic acid (LA), C18:2, µg/ml								
LCD	675.80 (99.95)	624.24 (97.09)	-48.08	-85.14 to -11.01	0.011	33.97	-16.43 to 84.36	0.186
Low-fat diet	648.04 (142.89)	564.98 (145.55)	-82.04	-120.44 to -43.65	**< 0.001**			
Gamma-linoleic acid (GLA), C18:3 n6, µg/ml								
LCD	10.45 (4.05)	4.37 (2.44)	-6.46	-7.84 to -5.07	**< 0.001**	-3.59	-5.50 to -1.69	**< 0.001**
Low-fat diet	10.74 (5.40)	7.47 (4.21)	-2.86	-4.33 to -1.40	**< 0.001**			
Eicosadienoic acid, C20:2, µg/ml								
LCD	5.70 (4.60)	3.07 (1.37)	-1.91	-5.94 to 2.13	0.355	-5.23	-10.35 to -0.10	0.046
Low-fat diet	4.20 (1.81)	8.24 (21.15)	3.32	-1.16 to 7.80	0.146			
Dihomo-gamma-linolenic acid (DGLA), C20:3, µg/ml								
LCD	43.2 (12.9)	20.3 (7.0)	-23.52	-27.37 to -19.68	**< 0.001**	-15.55	-20.72 to -10.38	**< 0.001**
Low-fat diet	42.04 (12.71)	34.19 (14.68)	-7.97	-11.88 to -4.07	**< 0.001**			
Arachidonic acid (AA), C20:4, µg/ml								
LCD	183.44 (40.84)	191.31 (36.96)	7.08	-2.42 to 16.59	0.144	-4.48	-17.79 to 8.84	0.510
Low-fat diet	172.38 (38.72)	184.62 (44.18)	11.56	1.66 to 21.46	0.022			
Adrenic acid, C22:4, µg/ml								
LCD	4.05 (1.31)	3.86 (1.08)	-0.27	-0.65 to 0.12	0.176	-0.13	-0.66 to 0.4	0.640
Low-fat diet	4.10 (1.16)	3.96 (1.35)	-0.14	-0.55 to 0.27	0.504			
Omega-3								
Alpha-linolenic acid (ALA), C18:3 n3, µg/ml								
LCD	20.60 (7.21)	15.69 (4.51)	-4.30	-6.35 to -2.24	**< 0.001**	-0.69	-3.47 to 2.09	0.628
Low-fat diet	18.04 (6.15)	14.87 (7.33)	-3.61	-5.76 to -1.46	**0.001**			
Eicosapentaenoic acid (EPA), C20:5, µg/ml								
LCD	18.15 (11.74)	13.14 (14.00)	-5.11	-7.58 to -2.63	**< 0.001**	-1.69	-5.17 to 1.79	0.342
Low-fat diet	15.53 (8.29)	12.15 (10.74)	-3.42	-6.00 to -0.85	**0.009**			
Docosapentaenoic acid (DPA), C22:5 n3, µg/ml								
LCD	11.50 (1.83)	9.36 (2.47)	-2.08	-2.85 to -1.31	**< 0.001**	0.30	-0.74 to 1.34	0.573
Low-fat diet	10.91 (2.30)	8.56 (2.84)	-2.38	-3.19 to -1.57	**< 0.001**			
Docosahexaenoic acid (DHA) C22:6, µg/ml								
LCD	45.01 (16.07)	48.48 (20.24)	3.63	-1.12 to 8.38	0.134	4.11	-2.53 to 10.76	0.225
Low-fat diet	42.63 (19.59)	44.27 (17.69)	-0.48	-5.49 to 4.52	0.850			

Data presented as mean (SD) and results from linear mixed models are presented as estimated marginal means (EMM), 95% confidence interval and *p *value. Significant *p* values are bolded. *N* = 35 in low-fat diet group at baseline (BL) and *n* = 28 at week 9 (w9). *N* = 33 in LCD group at BL and *n* = 32 at week 9. *CI* Confidence interval, *LCD* Low carbohydrate diet

Plasma concentrations of the MUFAs before and after the two diets, as well as changes within groups and differences between groups are presented in Table 2. Only the LCD group experienced a significant reduction in palmitoleic acid (-15.90 µg/mL), a reduction that was significantly larger compared to that observed in the low-fat diet group (-19.24 µg/mL). No changes were found in the low-fat diet group over time, or differences between groups for other MUFAs.

Plasma concentrations of the PUFAs before and after the two diets, as well as changes within groups and differences between groups are presented in Table 2. A significant decrease in GLA (-6.46 µg/mL and 2.86 µg/mL, respectively) and DGLA (-23.52 µg/mL and − 7.97 µg/mL, respectively) was seen in both the LCD and low-fat diet groups, however the reduction was greater in the LCD group compared to the low-fat diet group for both GLA (-3.59 µg/mL) and DGLA (-15.55 µg/mL). Only the low-fat diet group experienced a reduction in LA (-82.04 µg/mL). For the omega-3 fatty acids, a reduction in ALA (-4.30 µg/mL and − 3.61 µg/mL, respectively), EPA (-5.11 µg/mL and − 3.42 µg/mL, respectively), and DPA (-2.08 µg/mL and − 2.38 µg/mL) was found in both diet groups, with no significant differences between them.

### Associations with pain

The association between changes in pain and changes in the plasma concentration of FAs after adjusting for weight loss and group are presented in Table 3. The SFAs myristic acid and palmitic acid were positively associated with changes in pain, with an increase in the plasma concentration of these FAs being associated with increases in pain perception over time.

**Table 3:** Associations between changes in pain now and changes in the plasma concentration of FAs

Fatty acids	Std. Coefficient	Coefficient	95% CI	*P* value	Adj. *P* value^1^
Saturated fatty acids (SFA)					
Lauric acid, C12:0, µg/mL	0.11	0.12	-0.20 to 0.43	0.460	0.577
Myristic acid, C14:0, µg/mL	0.41	0.08	0.03 to 0.13	**0.002**	**0.044**
Palmitic acid, C16:0, µg/mL	0.37	0.01	0.00 to 0.01	**0.004**	**0.044**
Stearic acid, C18:0, µg/mL	0.30	0.02	0.00 to 0.03	0.023	0.128
Arachidic acid, C20:0, µg/mL	0.12	0.09	-0.12 to 0.31	0.391	0.538
Behenic acid, C22:0, µg/mL	0.05	0.01	-0.03 to 0.05	0.715	0.749
Lignoceric acid, C24:0, µg/mL	0.13	0.05	-0.06 to 0.16	0.336	0.493
Monounsaturated fatty acids (MUFA)					
Myristoleic acid, C14:1, µg/mL	0.27	0.55	-0.20 to 1.30	0.144	0.264
Palmitoleic acid (PA), C16:1, µg/mL	0.37	0.03	0.01 to 0.06	**0.009**	0.070
Oleic acid (OA), C18:1, µg/mL	0.24	0.00	-0.00 to 0.01	0.083	0.182
Eicosenoic acid, C20:1, µg/mL	-0.06	-0.01	-0.04 to 0.03	0.659	0.724
Nervonic acid, C24:1, µg/mL	-0.04	-0.01	-0.00 to 0.01	0.784	0.784
Polyunsaturated fatty acids (PUFA)					
Omega-6					
Linoleic acid (LA), C18:2, µg/mL	0.27	0.00	0.00 to 0.01	0.039	0.143
GLA, C18:3, µg/mL	0.28	0.12	-0.01 to 0.25	0.064	0.175
Eicosadienoic acid, C20:2, µg/mL	0.09	0.01	-0.03 to 0.05	0.499	0.578
GDLA, C20:3, µg/mL	0.33	0.05	0.00 to 0.09	0.039	0.143
Arachidonic acid (AA), C20:4, µg/mL	0.09	0.01	-0.01 to 0.03	0.479	0.578
Adrenic acid, C22:4, µg/mL	0.29	0.52	-0.03 to 1.07	0.064	0.175
Omega 3					
Alpha-linolenic acid (ALA), C18:3n3, µg/mL	0.15	0.05	-0.03 to 0.13	0.251	0.425
Eicosapentaenoic acid (EPA), C20:5, µg/mL	0.20	0.05	-0.02 to 0.12	0.131	0.263
Docosapentaenoic (DPA), C22:5 n3, µg/mL	0.14	0.12	-0.10 to 0.34	0.295	0.463
Docosahexaenoic acid (DHA) C22:6, µg/mL	0.24	0.03	-0.00 to 0.07	0.073	0.179

Data analyzed using multiple regression, with change in pain as dependent variable, and change in fatty acids as independent variables, after adjusting for group and magnitude of weight loss. Significant *p* values are bolded. Only coefficients from the fatty acids are presented. *CI* Confidence interval, *GDLA* Gihomo-gamma-linolenic acid, *GLA* Gamma-linolenic acid, *Std*. Standardized

^1^*P* value adjusted for Benjamini–Hochberg false discovery rate

## Discussion

The primary objective of this paper was to compare changes in the plasma concentrations of FAs between two low-energy diets; low-carbohydrate vs. low-fat diet, in females with lipedema and obesity. Secondary objectives were to investigate associations between changes in pain and changes in the plasma concentration of FAs. A reduction in the plasma concentration of several FAs was found, regardless of diet group. The SFAs myristic acid and palmitic acidwere significantly associated with changes in pain after adjusting for weight loss and diet group. One unit increase in the plasma concentration of myristic acid (1 µg/mL), was associated with a 0.1 cm increase in pain perception. In the present analysis, the plasma concentration of myristic acid decreased by a mean value of 11.3 µg/mL in the LCD group, which would then be expected to lead to a change in pain of one unit on a numeric rating scale from zero to ten. This represents a potentially meaningful change in pain, and suggests that LCDs may be more effective in reducing lipedema-associated pain.

Most FAs were significantly reduced regardless of diet group, due to the reduced energy intake, except for lignoceric acid that increased in the low-fat diet only, and this was significantly different from the LCD group. This increase in lignoceric may be due to increased endogen production of FAs, which may have occurred as a compensation to the reduced fat intake [26].

In this study, participants did not follow a strictly defined dietary fat composition. However, the LCD was planned to increase the intake of PUFAs while reducing SFAs. In contrast, the low-fat diet aimed to adhere to the Nordic Nutrition Recommendations [27]. To more precisely investigate the potential associations between plasma FA composition and pain in females with lipedema and obesity, future studies should consider randomizing participants to diets with distinct FA profiles, such as omega-3 supplementation, or designing interventions that target specific omega-6 to omega-3 FA ratios.

Changes in the SFA myristic acid were significantly associated with pain, and this FA were significantly reduced in the LCD, and to a greater extent than in the low-fat diet group. This may be due to the energy restriction, as well as the weak correlation observed between intake and the plasma concentrations of several FAs [20]. Especially the plasma concentrations of SFAs are rarely correlated with actual intake [20].

The SFA palmitic acid was significantly associated with pain after adjusting for weight loss and group, although no significant change within group or difference between groups were seen. This suggests that individual variability in palmitic acid changes may be related to individual variability in pain change. Despite not statistically significant, there was a trend towards a reduction in palmitic acid in the LCD (*P* = 0.016), potentially as a result of power restraint. The role of palmitic acid in pain may be partially explained through its pro-inflammation effects [28].

FA composition may reduce pain through reduced inflammation [29]. However, we did not find any differences between groups in inflammatory markers in this population [12]. Even though FA composition seems to be associated with changes in pain in this current paper, the lack of association between pain and inflammation may be due to the markers analyzed [12]. Relation with prostaglandins, bradykinin and leukotrienes should be investigated in future studies [30–32]. Several of the markers previously associated with lipedema, such as VEGF-A [33], VGF-C [34], IL-11, IL-28 A and IL-29 [35] were out of range or not included in the analysis of the present study. Additionally, the study may not be powered to detect differences between groups in inflammatory markers. Larger studies would be needed to investigate the association between plasma FA concentrations and inflammation in females with lipedema and obesity.

An animal study showed that SFAs can modulate pain, with palmitic or stearic acid increasing pain and lauric and myristic acid decreasing it [19]. The observed association with palmitic acid in the present study is consistent with these findings. However, the direction of association for myristic acid contradicts the previous animal data. This discrepancy may be attributable to differences in study design, particularly the dietary intervention. Whereas the referenced animal study employed diets with specifically controlled FA compositions, the present study utilized a broader dietary intervention (low-carbohydrate vs. low-fat), which may have influenced the FA profile and its effects on pain differently. Moreover, the underlying mechanism differs between humans and animals.

In human studies, most associations with pain are found for PUFAs [36], which was not the case in this present study. This may be due to the natural decrease in all FAs due to energy restriction. Also, the diets were not specifically designed to contain specific amounts of FAs, neither omega-3, nor omega-6. The LCD consisted of several dishes of fatty fish, although they were not encouraged to choose these meals over other meals.

The pathophysiology behind lipedema pain has not yet been determined, however several mechanisms have been suggested. Nociceptive pain and cutaneous allodynia [4, 5], as well as inflammation in the tissue [37] have been suggested. However, recently Dinnendahl et al. [6] suggested that the pain is not related to systemic or local inflammation, but a mechanism for pressure transmission caused by fibrosis in the extracellular matrix [6]. FA composition has been associated with fibrotic changes, more specifically SFAs, such as palmitic, myristic, lauric, stearic and behenic acid in patients with non-alcoholic steatohepatitis [38]. Palmitic and myristic acid were associated with pain in this present study. Our findings combined with the lack of association between pain and systemic inflammation [12], as well as the findings from Dinnendahl et al. [6], together suggest that FA composition may be associated with pain through reduction in fibrosis rather than inflammation. Considering we did not measure fibrosis in the tissue in this study, future studies should investigate the association between fibrosis in the lipedema tissue and pain following dietary interventions.

The present study has both strengths and limitations. This is the first study that has explored changes in plasma concentrations of FAs in females with lipedema following a low-carbohydrate versus a low-fat diet. Participants were randomized with stratification based on BMI categories, ensuring that BMI classes were equally distributed between the two diet groups. Despite the diets being matched for energy, a larger weight loss was seen in the LCD group. However, we adjusted for weight loss in our analyses. This is an explorative and secondary analysis of a randomized controlled trial, and the study was only powered to investigate pain. A larger number of statistical tests were performed and even though a lower significance level (0.01) was applied, the results must be interpreted with caution.

## Conclusion

A reduction in most FAs was found after energy restricted LCD and low-fat diets in females with lipedema and obesity. Reductions in SFAs seem to be associated with the reduction in pain seen in the LCD group, especially myristic acid. These findings suggest that FA composition may be important in relation to pain reduction in females with lipedema.

## Supplementary Information

Supplementary Material 1.

## Data Availability

Data described in the manuscript will be available upon request.

## Abbreviations

ALA: Alpha-linolenic acid
BL: Baseline
BMI: Body mass index
CHO: Carbohydrates
CI: Confidence interval
DGLA: Dihomo-gamma-linolenic acid
DHA: Docosahexaenoic acid
DPA: Docosapentaenoic acid
E%: Energy percentage
EPA: Eicosapentaenoic acid
FA: Fatty acid
GC: Gas chromatography
GC-FID: Gas chromatography with flame ionization detector
GLA: Gamma-linolenic acid
LCD: Low-carbohydrate diet
LMM: Linear mixed models
NTNU: Norwegian University of Science and Technology
OA: Oleic acid
PA: Palmitoleic acid
QC: Quality control
RCT: Randomized controlled trial
REK: Regional ethical Committee
SD: Standard deviation
SFA: Saturated fatty acid
w9: Week 9
AA: Arachidonic acid
