# Supplementary material for: Changes in plasma fatty acid composition in females with lipedema following low-carbohydrate vs low-fat diets and associations with pain reduction
Source: Nutr J. 2026 Mar 11;25:47. doi: 10.1186/s12937-026-01304-y (PMC13088385; doi:10.1186/s12937-026-01304-y)
Supplement: Supplementary file 1 — Supplementary Material 1. [file 12937_2026_1304_MOESM1_ESM.docx]

| Supplementary Table 1. Reported fatty acid intake from pre-coded food diaries. | | | | | | | | | |
| --- | --- | --- | --- | --- | --- | --- | --- | --- | --- |
|  |  | **Baseline** | **Week 9** | **Change from baseline to week 9** | | | **Difference in change between groups** | | |
|  |  | **Mean (SD)** | **Mean (SD)** | **EMM** | **95% CI** | **P value** | **EMM** | **95% CI** | **P value** |
| Total fats |  |  |  |  |  |  |  |  |  |
|  | LCD | 85.3 (26.4) | 62.8 (31.4) | -30.6 | -43.6 to -17.6 | **<0.001** | 27.1 | 11.5 to 42.7 | **0.001** |
|  | Low-fat diet | 102.3 (39.0) | 35.7 (9.4) | -57.7 | -71.2 to -44.2 | **<0.001** |  |  |  |
| Saturated fatty acids (SFA), g/day | |  |  |  |  |  |  |  |  |
|  | LCD | 33.5 (11.1) | 21.2 (10.5) | -16.6 | -22.1 to -11.0 | **<0.001** | 5.8 | -0.9 to 12.5 | 0.091 |
|  | Low-fat diet | 42.8 (18.4) | 15.8 (4.7) | -22.4 | -28.1 to -16.6 | **<0.001** |  |  |  |
| Monounsaturated fatty acids (MUFA), g/day | | |  |  |  |  |  |  |  |
|  | LCD | 32.0 (11.6) | 23.0 (15.6) | -11.2 | -16.8 to -5.7 | **<0.001** | 11.5 | 4.8 to 18.1 | **0.001** |
|  | Low-fat diet | 36.5 (15.1) | 11.4 (3.2) | -22.7 | -28.5 to -16.9 | **<0.001** |  |  |  |
| Polyunsaturated fatty acids (PUFA), g/day | | |  |  |  |  |  |  |  |
|  | LCD | 13.0 (5.5) | 13.0 (8.6) | -0.7 | -3.5 to 2.1 | 0.643 | 8.5 | 5.2 to 11.9 | **<0.001** |
|  | Low-fat diet | 14.4 (7.2) | 4.5 (2.0) | -9.2 | -12.1 to -6.3 | **<0.001** |  |  |  |
| OMEGA 3, g/day |  |  |  |  |  |  |  |  |  |
|  | LCD | 2.3 (1.0) | 2.7 (1.8) | -0.0 | -0.8 to 0.8 | 0.964 | 1.8 | 0.8 to 2.8 | **<0.001** |
|  | Low-fat diet | 3.1 (3.0) | 0.9 (0.6) | -1.8 | -2.7 to -1.0 | **<0.001** |  |  |  |
| OMEGA 6, g/day |  |  |  |  |  |  |  |  |  |
|  | LCD | 10.7 (4.2) | 10.3 (6.7) | -0.7 | -2.8 to 1.4 | 0.520 | 6.3 | 3.7 to 8.9 | **<0.001** |
|  | Low-fat diet | 11.2 (5.2) | 4.0 (1.8) | -7.0 | -9.2 to -4.8 | **<0.001** |  |  |  |
| OMEGA-6/OMEGA-3 RATIO | |  |  |  |  |  |  |  |  |
|  | LCD | 4.9 (1.6) | 4.3 (1.3) | -0.6 | -1.5 to 0.3 | 0.202 | -1.0 | -2.1 to 0.0 | 0.058 |
|  | Low-fat diet | 4.8 (2.4) | 5.3 (2.7) | 0.5 | -0.5 to 1.4 | 0.340 |  |  |  |
| Data presented as mean (SD) and results from linear mixed models are presented as estimated marginal means (EMM), 95% confidence interval and p value. N=29 in low-fat diet group at baseline (BL) and n=26 at week 9 (w9). N=32 in LCD group at BL and n=29 at week 9. CI: Confidence interval. LCD: low carbohydrate diet. | | | | | | | | | |
